# Date Seeds as a Natural Source of Dietary Fibers to Improve Texture and Sensory Properties of Wheat Bread

**DOI:** 10.3390/foods9060737

**Published:** 2020-06-04

**Authors:** Fatma Bouaziz, Amal Ben Abdeddayem, Mohamed Koubaa, Raoudha Ellouz Ghorbel, Semia Ellouz Chaabouni

**Affiliations:** 1 Enzyme Bioconversion Unit (UR13ES74), National School of Engineering P.O. Box 1173-3038, Sfax University, Sfax 3038, Tunisia; fatma.bouaziz22@yahoo.fr (F.B.); abdeddayemamal@gmail.com (A.B.A.); raoudha.ghorbel@enis.rnu.tn (R.E.G.); semia.chaabouni@enis.rnu.tn (S.E.C.); 2 Ecole Supérieure de Chimie Organique et Minérale, Université de Technologie de Compiègne, EA 4297 TIMR, 1 allée du réseau Jean-Marie Buckmaster, 60200 Compiègne, France; 3 Common Service Unit of Bioreactor Coupled with an Ultrafilter, National School of Engineering P.O. Box 1173-3038, Sfax University, Sfax 3038, Tunisia

**Keywords:** date seed, water-soluble polysaccharides, hemicellulose, bread formulation, alveographic analysis, bread quality

## Abstract

The aim of this work was to investigate the effect of date seed water-soluble polysaccharides (DSP) and hemicellulose (DSH) as dietary fiber sources in enhancing the wheat bread’s quality. DSP and DSH were extracted from the three date seed varieties Deglet Nour, Ghars Souf, and Allig. The extraction yields ranged from 3.8% to 6.14% and from 13.29% to 18.8%, for DSP and DSH, respectively. DSP and DSH showed interesting functional properties and were incorporated at 0.5% and 0.75% (*w/w*) in wheat flour with low bread-making quality (FLBM). The results showed that the addition of 0.75% DSH significantly improved the alveograph profile of the dough, and in a more efficient way than that of DSP. Furthermore, bread evaluation revealed that the addition of DSH considerably improved the volume (by 24.22%) and the texture profile of bread (decrease of the hardness and chewiness by 41.54% and 33.81%, respectively), compared to control bread (prepared with FLBM). A sensory analysis showed that the better overall acceptability was found for bread supplemented with DSH. Results in this work demonstrate that hemicellulose fraction extracted from date seeds (DSH) and added with a level of 0.75% to FLBM represents the component that improved bread quality the best.

## 1. Introduction

Date palm (*Phoenix dactylifera* L.) is one of the oldest plants cultivated by mankind and is broadly distributed worldwide, but mainly in the arid and semiarid regions [1,2]. Its fruit plays an important role in the economic and social life of people and is composed of a fleshy part (constituted of pericarp, epicarp, and mesocarp), and one seed (also called pit, kernel, or pyrene). The world’s date production was estimated to 8.52 million tons in 2018 [3], with approximately 852,000 tons of date seeds that are produced (i.e., considering 10% of the total fruit mass) and which can substitute some dietary fibers currently used. More than 4 million date palm trees are growing in Tunisia, which produce annually around 100,000 tons of fruits [4]. Date palm seeds are a waste product (or a by-product) derived from the technological transformations of the date fruits [5]. They represent up to ~15% of the date fruit’s weight, which leads to a large quantity of date seeds in the processing units [6,7]. Date seeds contain a high amount of fibers (75–80%), fats (10–12%), and proteins (5–6%) [8,9,10]. Carbohydrates composing date seeds are mainly of insoluble fiber types. For instance, the content of cellulose and hemicellulose in Deglet Noor date seeds are of ~50% and ~20%, respectively [11]. Despite their high fiber content, date seeds are still considered a by-product and are usually disposed of or used as animal feed in certain countries. The use of date seeds in animal feeds was first performed for dairy cows [12]. Other studies were conducted to valorize the date seeds to feed broiler [13], goats [14], and fish [15]. It was demonstrated that the date seeds used in animal feed enhanced the growth and increased the plasma level of estrogens [16] and testosterone [17]. Besides their high fiber content, date seeds are a great source of nutritive substances. In fact, date seeds contain high levels of phenolic compounds (21.0–62.0 mg gallic acid equivalents (GAE)/100 g date seeds), and antioxidants (580–929 mL Trolox equivalents/g date seeds) [18,19]. Therefore they have a great potential to be used as a supplement for antioxidants in nutraceutical, pharmaceutical, and medicinal products [1]. Supplementing date seeds to the human diet has been shown through a few works in the literature. For instance, defatted date seed powder was added to the wheat dough to replace wheat flour in bread at 1% and 3% replacement levels [11]. Significant changes in dough performance and bread quality were observed. In a more recent work, different amounts of date seed powder (0%, 1.5%, 3%, and 6%) were added to beef burgers, and its effect on the safety and quality was evaluated during 10 days storage [20]. The results showed improved shelf life and cooking properties of the burgers.

The development of staple foods enriched with fibers is thereby an important contribution to a broader supply of food products with beneficial health effects. Regular consumption of fiber is an important factor to prevent several diseases and is associated with a standard balanced diet [21]. Although a few works have demonstrated the impact of date seeds to supplement the human diet, no work has been performed to study the impact of date seed constituents on wheat dough. In this line, this work aims to investigate the effect of water-soluble polysaccharides (DSP) and hemicellulose (DSH) derived from date seeds on bread formulation. The alveograph characteristics of wheat flour dough were studied. The impact of using date seed derivatives as bread additives in terms of volume, texture, color, and sensory properties of the formulated bread was investigated.

## 2. Materials and Methods 

### 2.1. Wheat Flour and Date Seeds

Commercial soft wheat flour of low bread-making quality (FLBM) with 13.2% moisture, 0.4% ash, and 9.8% protein content was used. Premium quality wheat flour of high bread-making quality (FHBM) was used as a reference (13.68% moisture, 0.5% ash, and 12% protein content). Locust bean gum (LBG) was used as a reference polysaccharide.

Date seeds of three cultivars (Deglet Nour, Ghars Souf, and Allig) were isolated from 50 kg of date fruits collected at the full ripening stage from the same origin (Douz, Tunisia). The obtained seeds were first washed with distilled water to remove any adhering date flesh and then oven-dried for 48 h at 50 °C. Date seeds from each variety were separately milled in a heavy-duty grinder to pass 1–2 mm screen. The powder was then stored at −20 °C until use.

### 2.2. Chemical Characterization of Date Seeds

Dry matter was determined according to Cunniff [22]. Total nitrogen content was determined by Kjeldahl’s method. Protein content was calculated using the general factor 6.25 [23]. Fat and sugar contents were determined according to The French Association for Standardization (AFNOR) [24] and Dubois et al. [25], respectively. Ash content was measured by sample combustion in a muffle furnace at 550 °C for 4 h and the mineral composition was determined using an Atomic Absorption Spectrophotometer ZEEnit700 (Analytik Jena, Saint-Aubin, France). The enzymatic-gravimetric method was used to determine the dietary fiber content [26]. The content of water-soluble polysaccharides was determined under reflux for 3 h with 100 mL distilled water according to the standard method of Technical Association of Pulp and Paper Industry (TAPPI) T207 om-93. Acid hydrolysis of dried date seed powder, followed by filtration, drying, and weighting was used to determine the insoluble lignin content according to the standard TAPPI T222 om-11. The sodium chlorite method was used to determine the holocellulose (cellulose + hemicellulose) content according to the standard TAPPI T257 om-09. The cellulose content was then determined by hydrolyzing the extracted holocellulose with sodium hydroxide solution according to the standard TAPPI T257 om09. The hemicellulose content was determined by subtracting the amount of cellulose from holocellulose content. The starch content was evaluated by the enzymatic colorimetric method as previously described [27]. The titratable acidity of the date seeds was determined according to the Tunisian standard NT 52.15 (1982). The acidity was determined by titration with a sodium hydroxide solution (0.1 M).

### 2.3. Morphological Study of Date Seeds

The morphology of the date seeds used in this work was evaluated using 10 seeds randomly taken from each variety. The dimensions (length and width) and weights of date seeds were then recorded.

### 2.4. Extraction of Soluble Polysaccharides and Hemicellulose from Date Seeds 

**Figure 1:**
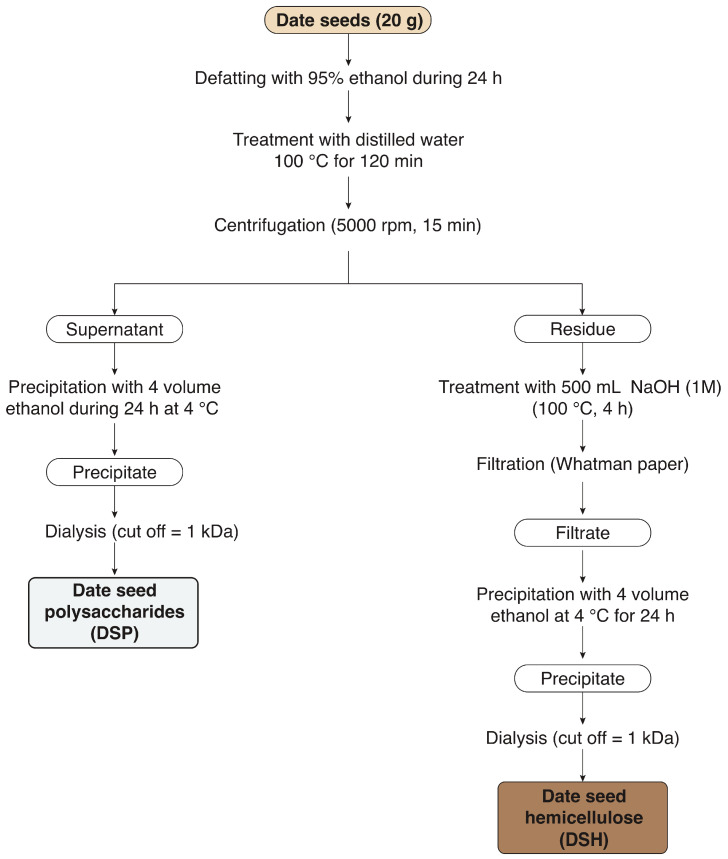
Extraction steps of the water-soluble polysaccharides (DSP) and hemicellulose (DSH) from the date seeds of the varieties Deglet Nour, Ghars Souf, and Allig.

The powdered date seeds were fractionated into water-soluble polysaccharides (DSP) and hemicellulose (DSH) according to Yao et al. [28] and Peng et al. [29], respectively, with slight modifications (Figure 1). The date seed powder obtained from each variety was first defatted with 95% ethanol during 24 h. After removing the impurities and lipophilic molecules, a 20 times dilution was performed on the defatted date seed powder followed by incubation at 100 °C in a thermostatic water bath for 120 min. The mixture was then filtered through a Whatman n° 4 filter paper, leading to obtain two fractions: a supernatant that was used to recover the DSP and a residue that served to recover the DSH. To precipitate the DSP, the supernatant was first concentrated 10 times under vacuum at 50 °C using a rotary evaporator (Shanghai, China), supplemented with 4 volumes of ethanol (96%), and then incubated for 24 h at 4 °C. The precipitated DSP were separated from the supernatant by centrifugation for 15 min at 3000× *g*. The supernatant was discarded and the precipitate (DSP) was re-suspended in deionized water. Minerals and low molecular weight molecules were removed from the extract during three days of dialysis (cut off = 1 kDa). Powdered DSP was obtained following a freeze-drying step and the recovery yield (%, *w/w*) was determined according to Equation (1).

(1) $$\mathrm{DSP}\;\mathrm{recovery}\;\mathrm{yield}\;\left(\%\right)=\frac{\mathrm{Lyophilized}\;\mathrm{DSP}\;\mathrm{weight}\;\left(g\right)}{\mathrm{Date}\;\mathrm{seed}\;\mathrm{powder}\;\mathrm{weight}\;\left(g\right)}\times 100$$

DSH was extracted on the other hand from the residue obtained at the first centrifugation step. The extraction was performed in basic conditions by adding 500 mL of NaOH (1 M) followed by incubation for 120 min at 100 °C in a thermostatic water bath. The subsequent steps of filtration, concentration, precipitation, dialysis, and freeze-drying were performed similarly to DSP. The DSH recovery yield (%, *w/w*) was determined according to Equation (2).

(2) $$\mathrm{DSH}\;\mathrm{recovery}\;\mathrm{yield}\;\left(\%\right)=\frac{\mathrm{Lyophilized}\;\mathrm{DSH}\;\mathrm{weight}\;\left(g\right)\;}{\mathrm{Date}\;\mathrm{seed}\;\mathrm{powder}\;\mathrm{weight}\;\left(g\right)}\times 100$$

### 2.5. Functional Properties of Date Seed Fractions

The functional properties (water and fat absorption capacities, emulsion properties, and foaming properties) of the date seed polysaccharides (DSP) and the date seed hemicellulose (DSH) obtained from the varieties Deglet Nour, Ghars Souf, and Allig were evaluated.

#### 2.5.1. Water-Holding Capacity (WHC)

The water-holding capacity of each fraction was determined as previously reported [30]. Mixtures of 0.5 g of DSP and DSH were prepared in 30 mL distilled water and kept at room temperature for 1 h. The resulting solutions were mixed for 5 s every 15 min. After 1 h, the solutions were centrifuged at 1600× *g* for 10 min and the supernatants were discarded. The centrifuge tubes were then drained for 30 min on a filter paper after tilting to a 45° angle. The water-holding capacity was calculated as gram of water retained by gram of sample on a dry basis, according to Equation (3).

(3) $$\mathrm{WHC}\;\left(g\;\mathrm{water}/g\;\mathrm{dry}\;\mathrm{sample}\right)=\frac{\;\mathrm{weight}\;\mathrm{of}\;\mathrm{the}\;\mathrm{tube}\;\mathrm{contents}\;\mathrm{after}\;\mathrm{draining}\;\left(g\right)-\mathrm{weight}\;\mathrm{of}\;\mathrm{dried}\;\mathrm{sample}\;\left(g\right)}{\mathrm{weight}\;\mathrm{of}\;\mathrm{dried}\;\mathrm{sample}\;\left(g\right)}$$

#### 2.5.2. Fat-Binding Capacity

The fat-binding capacity (FBC) was measured as previously reported [31], with modifications. The date seed samples (0.5 g of DSP and DSH) were mixed each with 10 mL corn oil and kept at room temperature for 30 min (vortexted for 5 s every 15 min). Afterwards, the suspension was centrifuged at 1600× *g* for 25 min and the supernatant was discarded. The centrifuge tube was then drained for 30 min on a filter paper after tilting to a 45° angle. The FBC was determined according to Equation (4).

(4) $$\mathrm{FBC}\;\left(g\;\mathrm{oil}/g\;\mathrm{dry}\;\mathrm{sample}\right)=\frac{\;\mathrm{weight}\;\mathrm{of}\;\mathrm{the}\;\mathrm{tube}\;\mathrm{contents}\;\mathrm{after}\;\mathrm{draining}\;\left(g\right)-\mathrm{weight}\;\mathrm{ofdried}\;\mathrm{sample}\;\left(g\right)}{\mathrm{weight}\;\mathrm{dried}\;\mathrm{sample}\;\left(g\right)}$$

#### 2.5.3. Emulsion Properties

Emulsion properties were determined as previously reported [32]. Different concentrations of DSP and DSH (0.25%, 0.5%, 1%, and 2% (*w/v*)) were prepared in 10 mL water and then mixed with 3 mL commercial corn oil. The mixtures were homogenized for 1 min at 20 °C using an Ultra Turrax homogenizer (IKA WERKE, Staufen im Breisgau, Germany) at 10,000 rpm. The suspensions were then centrifuged at 8000× *g* for 10 min. The emulsion capacity (EC) was calculated according to Equation (5).

(5) $$\mathrm{EC}=\left(\frac{V_{f}}{V_{i}}\right)\times 100$$

where V_f_ and V_i_ correspond respectively to the emulsion volume and the total volume.

The emulsion stability (ES) was determined in an emulsion that was kept motionless at room temperature for 30 min and then centrifuged at 8000× *g* for 10 min. ES was calculated according to Equation (6).

(6) $$\mathrm{ES}=\left(\frac{V_{t}}{V_{i}}\right)*100$$

where V_i_ corresponds to the initial emulsion volume and V_t_ to the final emulsion volume after incubation and centrifugation.

#### 2.5.4. Foaming Properties 

The foam capacity (FC) and foam stability (FS) of the DSP and DSH were determined as previously described [33]. Different concentrations of DSP and DSH (0.5%, 1%, 2%, and 4% (w/v)) were prepared in 20 mL water and then homogenized for 1 min at 10,000 rpm and 20 °C to incorporate air using an Ultra Turrax homogenizer (IKA WERKE, Staufen im Breisgau, Germany). The whipped samples were immediately transferred after homogenization into a graduated cylinder, and the total volume was measured before and after keeping the suspensions motionless for 30 min at room temperature. The FC was expressed as the percentage of volume increase compared to the state immediately after homogenization and was calculated according to Equation (7).

(7) $$\mathrm{FC}\;\left(\%\right)=\left(\frac{V_{T}-V_{0}}{V_{0}}\right)\times 100$$

The FS was calculated after keeping the suspensions motionless for 30 min according to Equation (8).

(8) $$\mathrm{FS}\;\left(\%\right)=\left(\frac{V_{t}-V_{0}}{V_{0}}\right)\times 100$$

where V_T_ is the total volume after whipping (mL), V_0_ is the volume before whipping, and V_t_ is the total volume after keeping the suspensions motionless for 30 min at room temperature.

### 2.6. Dough Preparation and Characterization

Dough samples were prepared by mixing the following ingredients: 300 g of wheat flour, 4.8 g of sucrose, 5.75 g of sodium chloride, 3 g of wet compressed yeast (*Saccharomyces cerevisiae*), 12 mL of corn oil, and 195 mL of water. The mixtures were kneaded, fermented and baked for 3 h in a bread maker (Home Carrefour HBM1228). The different ingredients were first put in the bread pan of the apparatus and kneaded for 14 min. The obtained dough was then fermented for 20 min before a second kneading for 20 min. The dough was left motionless at room temperature for 30 min after kneading.

Dough prepared with wheat flour of high bread-making quality (FHBM) was used as a positive control. Dough prepared with wheat flour of low bread-making quality (FLBM) was used as the negative control. Two amounts (0.5% and 0.75%) of date seed polysaccharides (DSP) and date seed hemicellulose (DSH), from the Allig variety (providing the highest extraction yields), were supplemented to FLBM. The DSP and DSH were used as additives to study their impact on the textural and nutritional properties of bread. Locust bean gum (LBG) was incorporated as a reference polysaccharide at the levels of 0.5% and 0.75%.

The alveographic properties of the dough samples were studied using an Alveograph Chopin (Châtillon, France). The following parameters were automatically recorded by a computer program developed by R Design company (Pullman, WA, USA): the maximum overpressure (P) needed to blow the dough bubble, which is an index of resistance to extension; the average abscissa (L) at bubble rupture, which is an index of dough extensibility; the deformation energy (W), which is an index of dough strength; and the P/L ratio (elastic resistance and extensibility balance of a flour dough), which indicates the dough quality.

### 2.7. Bread Making and Characterization

#### 2.7.1. Bread Making

Bread baking was performed in the bread pan of a bread maker (Home Carrefour HBM1228) for 60 min. After baking, the bread samples were cooled for 30 min before characterization.

#### 2.7.2. Bread Volume

The bread volume and weight were determined according to Yi et al. [34]. After baking and cooling, bread obtained from each mixture was first placed in a 2 L beaker (known volume, V_C_). The container was then topped with rapeseed and the bread was removed to record the volume of rapeseed, V_R_. The difference between the container volume and the rapeseed volume, V_C_–V_R_, gave the bread volume, V_B_. The bread weight, W_B_, was then recorded and the bread specific volume (V_S_) was calculated according to Equation (9):

(9) $$V_{s}\;\left({\mathrm{cm}}^{3}/g\right)=\frac{V_{B}}{W_{B}}$$

#### 2.7.3. Bread Textural Analysis

The bread texture was evaluated by means of texture profile analysis (TPA) test for each bread sample using a texturometer (Lloyd instruments ltd, Bognor Regis, England) equipped with a cylindrical aluminum probe of 35 mm diameter. The texture analyzer was interfaced with a computer, which controls the parameters and analyzes the data using software supplied by Texture Technologies Corp. (Scarsdale, NY, USA). The measurements were performed on bread pieces of 2 cm width, 4 cm length, and 5 cm height, by compressing to 40% of their original height. The parameters applied were of 0.5 mm·s^−1^ speed and 5 s delay time between two subsequent compressions. Bread slices were placed vertically under the probe. The textural curves obtained for each bread type were used to calculate the hardness, cohesion, and springiness (primary parameters), as well as the adhesion and chewiness (secondary mechanical characteristics) [35]. Regarding the primary parameters, the peak force obtained during the first compression cycle corresponds to the hardness, the ratio between the active work done under the second force-displacement curve and that done under the first compression curve corresponds to the cohesiveness (a dimensionless parameter), and the distance of the sample recovered after the first compression (mm) corresponds to the springiness. Concerning the secondary mechanical characteristics, the negative work necessary to pull the compressing plunger away from the sample corresponds to the adhesion (N), whereas the product of hardness, cohesiveness, and springiness provides the chewiness (N mm). All texture measurements were performed in triplicate for each bread sample. Besides the primary parameters and the secondary mechanical characteristics, the bread texture analyses were performed after bread cooling to room temperature (after ~2 h) [36].

#### 2.7.4. Bread Color Evaluation

The colors of the crumb and crust of bread were determined by measuring the CIE (International Commission on Illumination) Lab coordinates (L *, a *, b *) using a Mini Scan XETM spectrophotocolorimeter (HunterLab Inc., Reston, VA, USA). In this coordinate system, L * corresponds to the lightness from black (0) to white (100), a * from green (−) to red (+), and b * from blue (−) to yellow (+). The results of bread color were expressed as the average of five measurements taken at different points of the samples and at room temperature. A standard white plate (L * = 93.68, a * = 0.69, and b * = 0.88) was used as a reference for the measurements.

#### 2.7.5. Bread Sensory Analysis

The bread samples were coded with three numbers and homogeneously presented in an anonymous way, as previously described [37]. Forty panelists composed of 15 males and 25 females from the staff members and students of the National School of Engineering (Sfax, Tunisia), aged between 23 to 50 years, were chosen randomly for bread sensory analysis. Uniformly cut bread slices of 30 × 20 × 10 mm size were served with water to the panelists. A five-point hedonic scale (1 for “disliked extremely” and 5 for “liked extremely”) was used to evaluate the prepared bread samples. Six coded bread samples were provided for the panelists to evaluate the appearance, crumb, taste, texture, odor, and color. The overall acceptability corresponded to the average value of the sensory properties given by the panelists.

### 2.8. Statistical Analysis

All the experiments and analytical measurements were performed in triplicate. The average values were analyzed statistically meaning the analysis of variance (ANOVA). SPSS software (version 17.0, SPSS Inc., Chicago, IL, USA) was used for analyses, and the differences between the treatments at 95% confidence level (*p <* 0.05) were considered as significant.

## 3. Results and discussion

### 3.1. Chemical Composition of Date Seeds

The chemical compositions of date seeds of the three varieties (i.e., Deglet Nour, Ghars Souf, and Allig) are shown in Table 1. The results showed low moisture contents in the date seed samples, ranging from 4.76% to 8.02%. The highest moisture content was recorded for the variety of Deglet Nour (8.02%), whereas the lowest was for the Allig variety (4.76%). These low moisture contents allow easy storage at room temperature for later use. Similar results were found by Hamada et al. [9] and Besbes et al. [10]. The protein and fat contents in the three varieties ranged from 2.48% to 2.62% and from 8.66% to 11.29%, respectively. These results concur with those previously found by Al-Farsi et al. [18]. Besides, high content in carbohydrates ranging from 76.47% to 83.32% was found in each date variety. For instance, the Allig variety showed the highest carbohydrates content with 83.32%, whereas the variety of Ghars Souf had the lowest ones with 76.47%. Likewise, high contents of dietary fiber ranging from 80.33% to 83.71%, including soluble and insoluble fiber, were recorded. Dietary fibers in date seeds are mainly composed of water-soluble polysaccharides, hemicellulose, cellulose, and lignin. The water-soluble polysaccharides, here DSP, ranged from 4.4% to 6.72% in the three date seed varieties. Higher content in hemicellulose was recorded with 31.97%, 34.29%, and 42.3%, for the varieties of Deglet Nour, Ghars Souf, and Allig, respectively. Similar to hemicellulose, high contents in cellulose ranging from 26.6% to 33.92% were recorded. Additionally, the lignin contents of the date seeds ranged from 21.2% to 24.06% in the three varieties. The highest lignin content (24.06%) was found for the variety of Allig, while the lowest (21.2%) was obtained for the variety of Deglet Nour. These results concur with those found by Chandra et al. [38] and Nabili et al. [39]. The starch contents in the date seeds ranged from 4.23% to 6.39%. The ash contents were of 2.32%, 1.81%, and 0.78% for Deglet Nour, Ghars Souf, and Allig varieties, respectively. These values concur with those reported by Besbes et al. [10]. The analysis of the mineral composition showed that the date seeds were mainly rich in sodium, calcium, and magnesium (Table 1).

### 3.2. Physical Properties of Date Seeds

Non-significant differences (*p >* 0.05) were observed in the dimensions (i.e., length, diameter) and weight of the date seeds between the three varieties (i.e., Deglet Nour, Ghars Souf, and Allig). Both seed diameters (0.76 ± 0.08, 0.74 ± 0.05, and 0.72 ± 0.04 cm, respectively) and lengths (2.51 ± 0.09, 2.49 ± 0.12, and 2.5 ± 0.19 cm, respectively) of the three varieties were similar to those observed for Bousthammi seeds [40], whereas higher seed weights were observed in the current work (0.898 ± 0.11, 1.03 ± 0.08, and 1.05 ± 0.13 g, respectively).

### 3.3. Extraction Yields of DSP and DSH 

The extraction yields of DSP and DSH from the three date seed varieties of Deglet Nour, Ghars Souf, and Allig were significantly different (*p <* 0.05). DSP yields were of 5.67 ± 0.19%, 3.8 ± 0.16%, and 6.14 ± 0.09%, respectively. These yields were lower than the ones extracted from potato peels with 29.85 ± 1.49% [41]. The DSH yields, on the other side, were of 13.29 ± 0.23%, 14.13 ± 0.21%, and 18.8 ± 0.2%, respectively. These yields were lower than those of leaves and tops of different sugarcane varieties, which were in the range of 20–34% [42]. Both DSP and DSH yields remain lower than the contents reported in Table 1, showing that the extraction was incomplete. However, the values are proportional to those reported in Table 1. In fact, the highest extraction yields of DSP and DSH were observed for the Allig variety, which presents the highest levels of soluble fibers and hemicellulose, respectively (Table 1).

### 3.4. Functional Properties of DSP and DSH 

#### 3.4.1. Water-Holding and Fat-Binding Capacities

The water-holding and fat-binding capacities are functional properties closely related to the final product’s texture due to the interactions with the different components. The results obtained showed higher water and oil retention capacities for DSH compared to DSP, for all the date seed varieties (Table 2). Non-significant differences were observed between the water-holding capacities (WHC) of the DSP extracted from the varieties of Deglet Nour, Ghars Souf, and Allig (2.7 ± 0.11, 2.6 ± 0.1, and 2.5 ± 0.2, respectively). These values were higher than those of the water-soluble polysaccharides extracted from either almond or pistachio juice processing by-products (1.95 ± 0.10 g (H_2_O)·g^−1^ sample and 1.46 ± 0.62 g (H_2_O)·g^−1^ sample, respectively). This difference could be related to the level of polar hydroxyl groups in the samples and hence their extent of hydrodynamic interactions. In fact, the peripheral polar groups of DSP molecules give varied interactions with water. The WHC of DSH ranged between 3.4 and 6.3 g (H_2_O)·g^−1^ sample. These values were comparable to that of almond gum hemicellulose with WHC of 6.3 ± 0.21 g (H_2_O)·g^−1^ dry weight [32].

The fat-binding capacity (FBC) of DSP were of 7.1 ± 0.4, 4.5 ± 0.3, and 5.9 ± 0.4 g (oil)·g^−1^ sample for the varieties of Deglet Nour, Ghars Souf, and Allig, respectively. The FBC of DSH, ranging between 8.6 and 12 g (oil)·g^−1^ sample, was higher than that of the DSP. These values were comparable to that of date flesh (9.75 g (oil)·g^−1^ sample) and higher than that reported for orange dietary fiber concentrate (1.27 g (oil)·g^−1^ sample) [43].

#### 3.4.2. Emulsifying Properties of DSP and DSH

**Figure 2:**
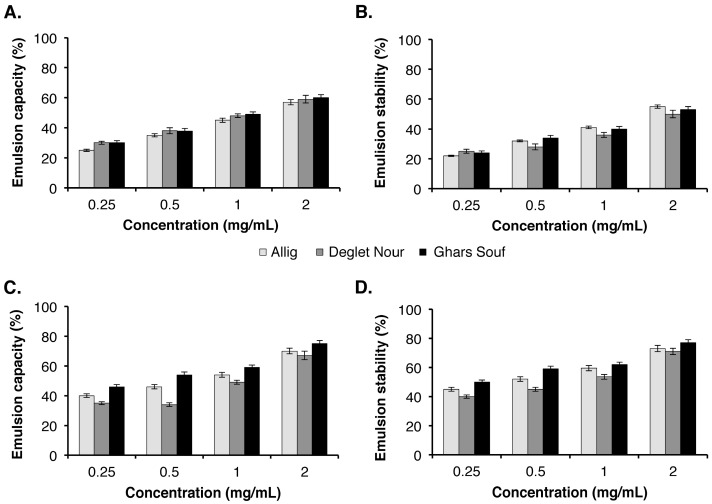
Emulsifying capacity and stability of DSP (**A**,**B**) and DSH (**C**,**D**) at different concentrations. Each value represents the average of three biological replicates ± standard deviation (SD). DSP, date seed polysaccharides; DSH, date seed hemicellulose.

The emulsion capacity (EC) and emulsion stability (ES) of DSP and DSH at different concentrations (0.25%, 0.5%, 1%, and 2% (*w/v*)) are presented in Figure 2. The results show that the EC values increased proportionally to the samples’ concentrations. However, the EC and ES values of DSH were significantly (*p* < 0.05) higher than those of DSP, regardless of the date variety and concentration. For instance, at 2% concentration, the EC values of DSH for the three date seed varieties ranged between 70% and 75%, whereas those of DSP ranged between 57% and 60%. The EC of DSP was significantly comparable for the three varieties (i.e., Allig, Deglet Nour, Ghars Souf) for the following concentrations 0.5%, 1%, and 2% (*w/v*). However, the EC of DSH from Ghars variety was significantly higher than the two other varieties for all the concentrations tested, and the EC of DSH from the Deglet Nour variety was the lowest. The obtained results demonstrate the great emulsifying ability of date seed derivatives. The EC of DSP for the three date seed varieties were lower than that of water-soluble polysaccharides isolated from almond juice processing by-products reaching an EC value of more than 90% at 4% concentration [44]. Additionally, the EC values of DSH of the three date seed varieties were lower than that reported for almond gum with EC values ranging from 85.28 ± 2% to 92.71 ± 1.6% at concentrations ranging from 0.5% to 3% [32].

#### 3.4.3. Foaming Properties of DSP and DSH

**Figure 3:**
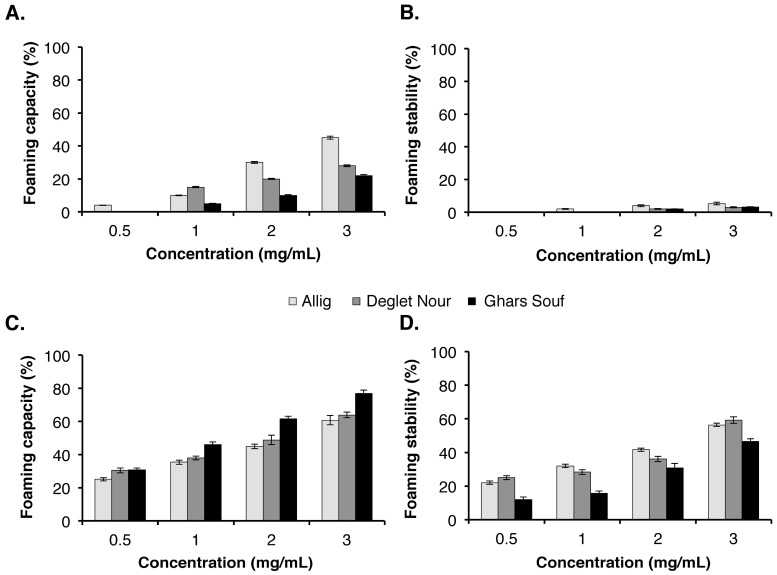
The foaming capacity and stability of the DSP (**A**,**B**) and DSH (**C**,**D**) at different concentrations. Each value represents the average of three biological replicates ± SD. DSP, date seed polysaccharides; DSH, date seed hemicellulose.

The foaming property is a surface property defined by its size and stability. Several food macromolecules, including proteins and polysaccharides, play an important role in foam stabilization [45]. They act by retarding liquid film drainage and by producing a viscoelastic layer at the bubble surface that protects the film against the rupture and prevents or retards the Ostwald ripening. Results of the foaming properties (i.e., foam capacity (FC) and foam stability (FS)) of DSP and DSH at different concentrations (0.5%, 1%, 2%, and 3% (*w/v*)) are shown in Figure 3. They indicate that the FC and FS increased proportionally to the sample’s concentration. The results show also that DSP does not form foam at a low concentration (0.5%), and has slight foam generation ability beyond 1% concentration, for the three date seed varieties. Besides, significant differences were observed between the FC values of DSP beyond 1% concentration. On the other side, the FC and FS values of DSH were significantly higher than those of DSP, regardless of the concentration used and the date seed variety. Indeed, even at the low concentration of 0.5%, the DSH form a stable foam. This behavior suggests that a high concentration of DSH could further improve the foam generation, which promotes the development of smaller and denser bubbles and increases the liquid retention in foams. Indeed, at 3% concentration, the average FC and FS values of DSH for the varieties of Deglet Nour, Ghars Souf, and Allig were of 63.93%, 76.92%, and 60.71%, and 59.2%, 46.5%, and 56.32%, respectively. The differences between the varieties could be related to the chemical composition of the fibers. The values obtained were higher than those of almond gum hemicellulose, which showed very low values of foam that disappeared after just 1 min of observation [32]. Moreover, these FC values were significantly higher than those obtained for polysaccharides extracted from potato peels (PPPW) in which the authors reported that at a concentration of 6%, the FC of PPPW was more than 50% [41]. These results indicate that DSH presents a promising potential to be used at different concentrations for the improvement of functional properties in different food formulations.

### 3.5. Alveographic Properties of Dough

The premium quality wheat flour of high bread-making quality (FHBM) was used as a positive control, and locust bean gum (LBG) was incorporated as reference polysaccharide. The DSP and DSH of the Allig variety were used in the alveograph and baking test as they provided significantly higher extraction yields than the two other varieties (i.e., Deglet Nour and Ghars Souf). The incorporation of DSP and DSH of the Allig variety to dough ingredients showed differences in the dough properties measured by the alveograph. The effect of DSP and DSH supplementation on the alveograph characteristics of wheat flour dough is shown in Table 3. The results obtained showed that the low wheat flour (FLBM) used was characterized by a low deformation work of 102 × 10**^−^**^4^ J and by a low P/L ratio (elastic resistance and extensibility balance of flour dough) of 0.5. Furthermore, the addition of DSH at a level of 0.75% to the FLBM significantly improved the values of the deformation work and the P/L ratio compared to the control, without DSH. In fact, the deformation energy increased to 194 × 10**^−^**^4^ J and the P/L ratio reached 1.28 after incorporating the DSH. The deformation work was similar to the premium flour (FHBM) and the P/L ratio was higher than that found for FHBM. The behavior of dough enriched with DSH was comparable to that supplemented with LBG. This effect is likely due to the interaction between DSH and wheat flour proteins, as previously reported [46]. While DSH incorporation significantly increased the dough quality, a slight improvement was observed by the addition of DSP, with a deformation work of 154 × 10**^−^**^4^ J and a P/L ratio of 0.42 (Table 3). Dietary fibers can impart some functional properties to foods (e.g., increase of the water-holding capacity, oil-holding capacity, emulsification and/or gel formation). Thus, dietary fibers incorporated in bakery products can modify the alveographic properties of dough and the textural properties of the bakery products. The difference between the DSP and DSH alveograph characteristics (W and P/L) could be related to the structure and composition of these fibers. Moreover, the formation of dough is an important step in the processing of flour products. The proper formation of a continuous network of wheat gluten imparts dough with both viscosity and elasticity. It has been reported that dough quality is directly governed by the gluten network structure. In fact, some authors have studied the effect of dietary fibers on dough properties and found that the gluten network structure was both improved and deteriorated by dietary fibers addition [47,48]. This behavior could be related to the differences in the type, structure, size, and amount of dietary fibers added [49].

### 3.6. Bread Characterization

**Figure 4:**
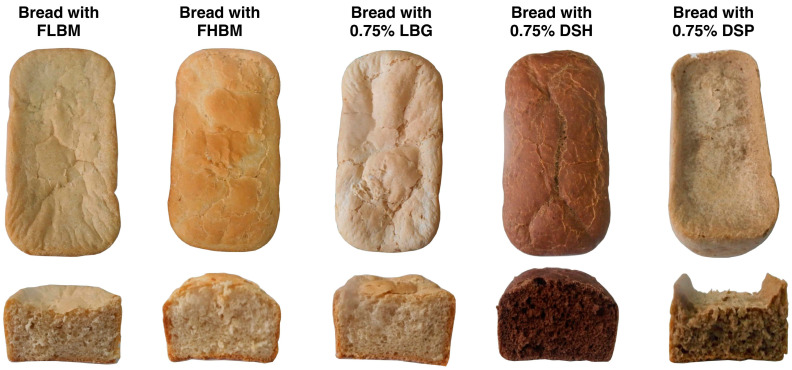
Bread photos (front view and cross-section) prepared with FLBM, FHBM, FLBM supplemented with 0.75% LBG, FLBM supplemented with 0.75% DSH from the Allig variety, FLBM supplemented with 0.75% DSP from the Allig variety. FLBM, flour of low bread-making quality; FHBM, flour of high bread-making quality; LBG, locust bean gum; DSH, date seed hemicellulose; DSP, date seed polysaccharides.

Based on the alveographic characteristics, dough supplemented with 0.75% DSP and DSH were baked and their characteristics were compared to those prepared only with FLBM and FHBM, and to that supplemented with 0.75% LBG. Bread photos are presented in Figure 4 and show that the morphological aspect is in agreement with the alveographic dough results. Indeed, the aspect of bread supplemented with DSH was more visually attractive than that prepared with the FHBM. On the other hand, bread prepared with FLBM and that supplemented with DSP have lower attractive appearance and lower volumes compared to reference bread, prepared with FHBM. Bread quality was evaluated by measuring the specific volume, texture, color, and by evaluating the sensory properties.

#### 3.6.1. Analysis of Bread Volume

**Figure 5:**
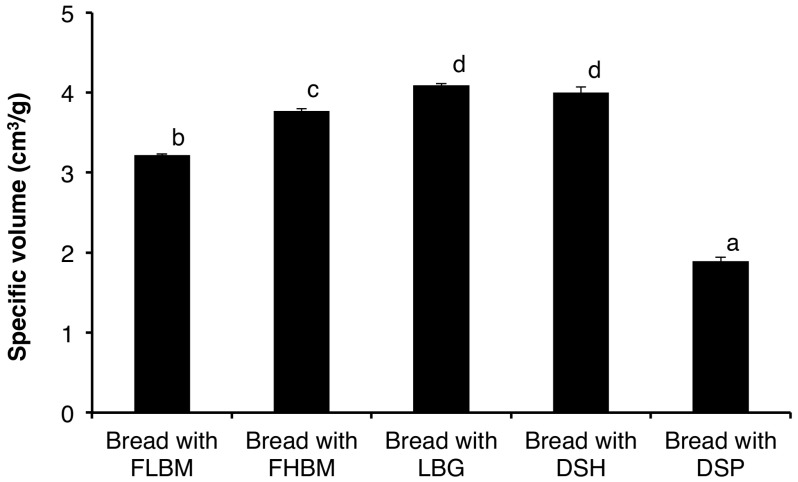
Specific volume (cm^3^/g) of bread prepared with FLBM, FHBM, FLBM supplemented with 0.75% LBG, FLBM supplemented with 0.75% DSH, and FLBM supplemented with 0.75% DSP. FLBM, flour of low bread-making quality; FHBM, flour of high bread-making quality; LBG, locust bean gum; DSH, date seed hemicellulose; DSP, date seed polysaccharides. The values not sharing the same letter (a–d) are significantly different (*p* < 0.05).

Loaf volume is considered the most important bread characteristic since it provides a quantitative measurement of baking performance [50,51]. For every bakery product, there is usually a relation between the dough weight and the loaf volume that yields the most desirable texture [34,52]. Figure 5 shows the specific volume of bread prepared using FLBM, FHBM, and FLBM supplemented with 0.75% DSP, 0.75% DSH, and 0.75% LBG. Bread prepared using either FLBM or FLBM supplemented with 0.75% DSP had the lowest specific volumes of 3.22 ± 0.01 cm^3^·g^−1^ and 1.89 ± 0.05 cm^3^·g^−1^, respectively. Nevertheless, bread supplemented with either 0.75% DSH or 0.75% LBG had the greatest specific volumes of 4 ± 0.07 and 4.09 ± 0.02 cm^3^·g^−1^, respectively. Many studies have focused on the addition of dietary fibers to baked products. It has been reported that dietary fibers could modify the bread loaf volume and the softness of the bread crumb [53,54]. The incorporation of fibers in bread can reduce or increase the loaf volume and this depends on the fiber source and the supplementation level. Indeed, bread enriched with wheat bran [55] or *β*-glucans [56] reduced the loaf volume and increased the crumb firmness of bread. However, bread enriched with gums such as almond gum, containing 72.7 ± 1.5% fibers, presented increased loaf volume. Beyond 2% almond gum concentration, bread volume decreased [11].

#### 3.6.2. Textural Properties of Bread

The values of the texture profile analyses (TPA) of bread prepared with FLBM, FHBM, and FLBM supplemented with DSP, DSH, and LBG are given in Table 4. The results obtained showed a great improvement of the textural properties of bread supplemented with DSH compared to that prepared with FLBM.

The hardness of bread supplemented with 0.75% DSH decreased significantly (*p* < 0.05) by 41.54% compared to control bread prepared with FLBM. Bread supplemented with DSH decreased its hardness, compared to bread prepared either with the reference flour (FHBM) or FLBM enriched with LBG. Moreover, the hardness of bread prepared with FHBM and bread supplemented with 0.75% LBG significantly (*p* < 0.05) decreased by 15.64% and 14.20%, respectively, compared to control bread prepared with FLBM. The hardness is mainly attributed to the amylose and amylopectin matrix, which contributes to the overall bread texture [57]. It was demonstrated that bread hardness is a result of the interactions between gluten and fibrous materials [58]. Likewise, Bouaziz et al. [11] showed that the addition of almond gum on bread formulation significantly reduced the hardness compared to control bread (without almond gum). Conversely, higher hardness values were measured for bread supplemented with 0.75% DSP, compared to control bread. This behavior is probably due to the presence of different interactions, compared to those made by LBG and DSH. Besides, the cohesiveness of bread supplemented with 0.75% DSH increased by 26.66%, whereas its springiness and adhesiveness were unchanged (*p* > 0.05) compared to control bread prepared with FLBM. Moreover, the chewiness of bread supplemented with 0.75% DSH and that prepared with the reference flour FHBM were significantly reduced by 33.81% and 72.2%, respectively, compared to control bread prepared with FLBM. Supplementing bread with 0.75% of DSP significantly increased (*p* < 0.05) the hardness by 25.71% compared to bread prepared with the control flour FLBM. Similarly, Chang et al. [59] showed that the incorporation of lemon fiber extracted from lemon pomace into bread increased the hardness proportionally to the fiber substitution level (0–9%). In addition, supplementing bread with 0.75% of DSP increased significantly (*p* < 0.05) the adhesiveness and the cohesiveness of bread by 75% and 45.45%, respectively, compared to bread prepared with the control flour FLBM.

#### 3.6.3. Analysis of Bread Color

Bread color results presented in Table 5 show that the crust and crumb lightness significantly decreased (*p* < 0.05) after the incorporation of DSP and DSH in bread formulation, compared to the control and reference flours. Indeed, the lightness (L*) values were of 63.25, 70.97, 29.55, and 43.26 for bread prepared with FLBM, FHBM, DSH, and DSP, respectively. Similar results were observed by the enrichment of wheat bread with pea and broad bean pod fibers [57]. Besides, the redness (a*) values of crust and crumb increased significantly (*p* < 0.05) after the incorporation of DSP and DSH, compared to control bread. Moreover, the yellowness (b*) of the bread’s crumb and crust supplemented with DSP and DSH decreased significantly (*p* < 0.05) compared to control bread. The darkness of the crumb is directly related to the quantity of fibers added (DSH or DSP). In fact, a significant decrease of the crust and crumb lightness and yellowness, and a significant increase of the redness were observed after the incorporation of DSP and DSH in bread. In addition, the residual proteins and carbohydrates in DSH and DSP probably contributed to the darker crust color through the development of Maillard reaction pigments. This reaction occurs between the free amino group of lysine and/or other amino acids and the carbonyl groups of reducing sugars [60]. Similar results were reported by Dalgetty and Baik [61] and Capuano et al. [60] when the crust of bread containing fibers was much darker than the other bread samples.

#### 3.6.4. Bread Sensory Evaluation

The effects of DSP and DSH incorporation on the sensory attributes (i.e., appearance, color, odor, taste, tenderness, and overall appreciation) of bread are shown in Table 6. The obtained results show that the bread supplemented with DSH and that prepared with the reference flour FHBM were appreciated significantly more (*p* < 0.05) by the panelists than that enriched with DSP, LBG, and prepared with the control flour FLBM. Indeed, the overall appreciation values of bread enriched with DSH and bread prepared with the reference flour FHBM were of 4.3 ± 0.12 and 4.55 ± 0.25, respectively. However, these values were of 3 ± 0.21, 2 ± 0.11, and 2.28 ± 0.2 for bread prepared with DSP, LBG, and the control flour FLBM, respectively (Table 6). Moreover, the appearance and the texture of bread supplemented with DSH and bread prepared with the reference flour FHBM were significantly more appreciated than the other prepared bread. Moreover, the taste and the color of the crumb and crust of bread supplemented with DSH and that prepared with FHBM were also appreciated by the panelists. In the literature, the sensory evaluation of various commercial fibers (carob fiber and inulin) used as fiber-enriching agents in bread making was reported and consumer panelists judged these fiber-enriched bread samples as acceptable [54]. Moreover, the sensory evaluation of the samples tested in this study was far superior to those obtained with other fibers, such as dietary fibers prepared from rice straw [53] and pea and broad bean pod fibers [57].

## 4. Conclusions

The date seed by-product is an important source of dietary fibers that could be valorized by incorporating their derivatives, especially the water-soluble polysaccharides (DSP) and hemicellulose (DSH) in baking. From the overall results, it can be concluded that the addition of DSH to wheat flour at different concentrations improves the rheological properties of the dough and the texture of enriched bread, in a more efficient way than that of DSP. Supplementing bread with 0.75% of DSH increased the deformation energy and the configuration curve ratio of dough. Furthermore, bread evaluation revealed that the addition of DSH decreased the hardness by 41.54% compared to control bread. In addition, bread supplemented with DSH and that prepared with the reference flour FHBM were appreciated significantly more (*p* < 0.05) by the panelists than the other prepared bread. Based on the results obtained, it can be concluded that DSH could be used as an agent for improving baking to further increase the daily fiber intake of the final product.

## Figures and Tables

**Table 1:** Chemical composition of the three date seeds varieties of Allig, Deglet Nour, and Ghars Souf.

	Allig Variety	Deglet Nour Variety	Ghars Souf Variety
Moisture (g·100 g^−1^)	4.76 ± 0.2 ^a^	8.02 ± 0.18 ^b^	7.81 ± 0.12 ^b^
Proteins (g·100 g^−1^)	2.48 ± 0.15 ^a^	2.49 ± 0.15 ^a^	2.62 ± 0.04 ^a^
Lipids (g·100 g^−1^)	8.66 ± 0.12 ^a^	9.77 ± 0.23 ^b^	11.29 ± 0.09 ^c^
Carbohydrates (g·100 g^−1^)	83.32 ± 0.19 ^c^	77.22 ± 0.15 ^b^	76.47 ± 0.22 ^a^
Insoluble fibers (g·100 g^−1^)	76.99 ± 0.21 ^b^	75.93 ± 0.12 ^a^	76.62 ± 0.09 ^b^
Soluble fibers (g·100 g^−1^)	6.72 ± 0.2 ^b^	4.40 ± 0.13 ^a^	6.23 ± 0.09 ^b^
Cellulose (g·100 g^−1^)	26.60 ± 0.08 ^a^	33.92 ± 0.07 ^c^	31.94 ± 0.09 ^b^
Hemicellulose (g·100 g^−1^)	42.30 ± 0.3 ^c^	31.97 ± 0.26 ^a^	34.29 ± 0.24 ^b^
Insoluble lignin (g·100 g^−1^)	24.06 ± 0.04 ^b^	21.20 ± 0.06 ^a^	23.96 ± 0.03 ^b^
Ash (g·100 g^−1^)	0.78 ± 0.08 ^a^	2.32 ± 0.08 ^b^	1.81 ± 0.72 ^b^
Zn (mg·100 g^−1^)	0.32 ± 0.01 ^b^	0.29 ± 0.02 ^a^	0.325 ± 0.01 ^b^
Mn (mg·100 g^−1^)	0.06 ± 0.01 ^a^	Trace	0.10 ± 0.008 ^b^
Fe (mg·100 g^−1^)	Trace	Trace	Trace
Cu (mg·100 g^−1^)	0.29 ± 0.01 ^a^	0.25 ± 0.01 ^a^	0.28 ± 0.01 ^a^
K (mg·100 g^−1^)	2.45 ± 0.1 ^b^	2.7 ± 0.21 ^b^	1.93 ± 0.14 ^a^
Na (mg·100 g^−1^)	5.86 ± 0.3 ^a^	5.65 ± 0.24 ^a^	5.41 ± 0.18 ^a^
Ca (mg·100 g^−1^)	5.51 ± 0.22 ^c^	3.12 ± 0.3 ^a^	3.63 ± 0.21 ^b^
Mg (mg·100 g^−1^)	2.57 ± 0.01 ^c^	1.98 ± 0.01 ^a^	2.13 ± 0.01 ^b^

Note: The data are the average values ± standard deviations of three replicates. The values not sharing the same letters (a–c) within a line are significantly different (*p* < 0.05).

**Table 2:** The water-holding and fat-binding capacities of the three date seed varieties of Deglet Nour, Ghars Souf, and Allig.

	Date Seed Variety	Water-Holding Capacity (g·g^−1^ Sample)	Fat-Binding Capacity (g·g^−1^ Sample)
DSP	Deglet Nour	2.7 ± 0.1 ^a^	7.1 ± 0.4 ^c^
	Ghars Souf	2.6 ± 0.1 ^a^	4.5 ± 0.3 ^a^
	Allig	2.5 ± 0.2 ^a^	5.9 ± 0.4 ^b^
DSH	Deglet Nour	6.3 ± 0.2 ^c^	12 ± 0.1 ^c^
	Ghars Souf	3.4 ± 0.2 ^a^	8.6 ± 0.2 ^a^
	Allig	5.6 ± 0.2 ^b^	9 ± 0.2 ^b^

Note: DSP, date seed water-soluble polysaccharides; DSH, date seed hemicellulose. The data are the means ± standard deviation values of three replicates. The different lowercase letters within a column indicate significant differences (*p* < 0.05).

**Table 3:** The alveograph characteristics of dough containing different levels of DSP and DSH extracted from the Allig variety.

	Deformation Work (10^−4^ J)	P/L Ratio
FLBM	102 ± 3.24 ^a^	0.45 ± 0.03 ^b^
FHBM	194 ± 3.75 ^e^	0.7 ± 0.02 ^d^
FLBM + 0.5% DSP	125 ± 2.91 ^b^	0.39 ± 0.03 ^a^
FLBM + 0.75% DSP	154 ± 2.35 ^d^	0.42 ± 0.01 ^ab^
FLBM + 0.5% DSH	153 ± 3.23 ^cd^	0.56 ± 0.03 ^c^
FLBM + 0.75% DSH	194 ± 2.41 ^e^	1.28 ± 0.02 ^f^
FLBM + 0.5% LBG	147 ± 2.35 ^c^	0.41 ± 0.02 ^a,b^
FLBM + 0.75% LBG	216 ± 3.25 ^f^	0.86 ± 0.03 ^e^

Note: DSP, date seed water-soluble polysaccharides; DSH, date seed hemicellulose; FLBM, flour of low bread-making quality; FHBM, flour of high bread-making quality; LBG, locust bean gum; P/L ratio, elastic resistance and extensibility balance of flour dough. All the values represent the average ± standard deviation of triplicate experiments. Different lowercase letters within a column indicate significant differences (*p* < 0.05) between the different fractions.

**Table 4:** The effect of DSP and DSH addition on the textural properties (hardness, cohesiveness, adhesiveness, springiness, and chewiness) of bread.

	Hardness (*N*)	Cohesiveness	Springiness (mm)	Adhesiveness (*N*)	Chewiness (Nmm)
Control bread with FLBM	5.56 ± 0.31 ^c^	0.11 ± 0.05 ^b^	11.84 ± 2.12 ^c^	0.52 ± 0.13 ^a^	7.887 ± 0.6 ^c^
Reference bread with FHBM	4.69 ± 0.2 ^b^	0.086 ± 0.007 ^a^	5.42 ± 0.65 ^a^	0.4 ± 0.02 ^a^	2.19 ± 0.15 ^a^
Bread with 0.75% LBG	4.77 ± 0.5 ^b^	0.11 ± 0.014 ^b^	12.67 ± 1.23 ^c^	0.84 ± 0.2 ^b^	9.82 ± 2.5 ^c^
Bread with 0.75% DSH	3.25 ± 0.32 ^a^	0.15 ± 0.014 ^c^	10.41 ± 0.0022 ^b,c^	0.5 ± 0.096 ^a^	5.22 ± 0.4 ^b^
Bread with 0.75% DSP	6.99 ± 0.65 ^d^	0.16 ± 0.016 ^c^	8.71 ± 1.2 ^b^	0.91 ± 0.013 ^b^	9.738 ± 1.46 ^c^

Note: DSP, date seed water-soluble polysaccharides; DSH, date seed hemicellulose; FLBM, flour of low bread-making quality; FHBM, flour of high bread-making quality; LBG, Locust bean gum. The data are the means ± standard deviation values of three replicates. The values not sharing the same letter (a–d) within a column are significantly different (*p* < 0.05).

**Table 5:** The effect of DSP and DSH addition on the color characteristics of bread.

	Control Bread with FLBM	Reference Bread with FHBM	Bread with 0.75% LBG	Bread with 0.75% DSH	Bread with 0.75% DSP
**Crumb**					
L*	63.25 ± 0.26 ^c^	70.97 ± 0.79 ^d^	60.24 ± 0.31 ^c^	29.55 ± 2.057 ^a^	43.26 ± 3.35 ^b^
a*	0.94 ± 0.1 ^a^	0.51 ± 0.03 ^a^	0.84 ± 0.04 ^a^	7.94 ± 0.54 ^c^	4.67 ± 0.28 ^b^
b*	18.13 ± 0.2 ^c,d^	18.94 ± 0.09 ^d^	17.58 ± 0.1 ^c^	8.57 ± 0.86 ^a^	12.20 ± 0.48 ^b^
**Crust**					
L*	68.52 ± 0.68 ^c^	67.39 ± 0.03 ^c^	64.23 ± 0.56 ^b^	35.59 ± 0.16 ^a^	63.37 ± 0.01 ^b^
a*	1.33 ± 0.05 ^b^	6.135 ± 0.02 ^d^	1.04 ± 0.032 ^a^	10.21 ± 0.03 ^e^	4.165 ± 0.06 ^c^
b*	20.65 ± 0.16 ^c^	29.85 ± 0.04 ^e^	22.56 ± 1.1 ^d^	11.73 ± 0.01 ^a^	16.59 ± 0.17 ^b^

Note: The data are the averages ± standard deviation of three replicates. The values not sharing the same letter (a–e) within a line are significantly different (*p* < 0.05). DSP, date seed water-soluble polysaccharides; DSH, date seed hemicellulose; FLBM, flour of low bread-making quality; FHBM, flour of high bread-making quality; LBG, Locust bean gum; L*, lightness; b*, yellowness; a*, redness.

**Table 6:** Sensory evaluation of the different prepared bread.

	Control Bread with FLBM	Reference Bread with FHBM	Bread with 0.75% LBG	Bread with 0.75% DSP	Bread with 0.75% DSH
Overall acceptability	2.28 ± 0.2 ^a^	4.55 ± 0.25 ^c^	2 ± 0.11 ^a^	3 ± 0.21 ^b^	4.3 ± 0.12 ^c^
Appearance	2.14 ± 0.4 ^a^	4.85 ± 0.74 ^b^	2.16 ± 0.2 ^a^	2.75 ± 0.32 ^a^	4.2 ± 0.5 ^b^
Odor	3.22 ± 0.14 ^b^	4.15 ± 0.58 ^c^	2.44 ± 0.3 ^a^	3.33 ± 0.12 ^b^	4.11 ± 0.7 ^c^
Texture	2.22 ± 0.5 ^a^	3.55 ± 0.22 ^b^	2.77 ± 0.17 ^a^	4 ± 0.5 ^b,c^	4.3 ± 0.22 ^c^
Taste	2.44 ± 0.12 ^a^	4.66 ± 0.36 ^b^	2.55 ± 0.56 ^a^	2.55 ± 0.74 ^a^	4.1 ± 0.31 ^b^
Crust color	2.55 ± 0.22 ^a^	4 ± 0.23 ^c^	2.88 ± 0.81 ^a,b^	3.44 ± 0.22 ^b,c^	3.8 ± 0.16 ^c^
Crumb color	2.44 ± 0.31 ^a^	3.66 ± 0.4 ^b^	2 ± 0.21 ^a^	3.77 ± 0.3 ^b^	3.66 ± 0.45 ^b^

Note: The data are the average ± standard deviation of three replicates. The values not sharing the same letter (a–d) within a line are significantly different (*p* < 0.05). DSP, date seed water-soluble polysaccharides; DSH, date seed hemicellulose; FLBM, flour of low bread-making quality; FHBM, flour of high bread-making quality; LBG, Locust bean gum.
